# The role of guidance in delivering cardiac resynchronization therapy: A systematic review and network meta-analysis

**DOI:** 10.1016/j.hroo.2022.07.005

**Published:** 2022-07-20

**Authors:** Vishal S. Mehta, Salma Ayis, Mark K. Elliott, Nadeev Widjesuriya, Nuha Kardaman, Justin Gould, Jonathan M. Behar, Amedeo Chiribiri, Reza Razavi, Steven Niederer, Christopher A. Rinaldi

**Affiliations:** ∗ Cardiology Department, Guy’s and St Thomas’ NHS Foundation Trust, London, United Kingdom; † School of Biomedical Engineering and Imaging Sciences, King’s College London, London, United Kingdom; ‡ School of Population Health and Environmental Sciences, King's College London, London, United Kingdom

**Keywords:** Cardiac resynchronization therapy, LV lead, Guidance, Efficacy, Systematic review, Meta-analysis

## Abstract

**Background:**

Positioning the left ventricular lead at the optimal myocardial segment has been proposed to improve cardiac resynchronization therapy (CRT) response.

**Objectives:**

We performed a systematic review and network meta-analysis evaluating echocardiographic and clinical response delivered with different guidance modalities compared to conventional fluoroscopic positioning.

**Methods:**

Randomized trials with ≥6 months follow-up comparing any combination of imaging, electrical, hemodynamic, or fluoroscopic guidance were included. Imaging modalities were split whether one modality was used: cardiac magnetic resonance (CMR), speckle-tracking echocardiography (STE), single-photon emission computed tomography, cardiac computed tomography (CT), or a combination of these, defined as “multimodality imaging.”

**Results:**

Twelve studies were included (n = 1864). Pair-wise meta-analysis resulted in significant odds of reduction in left ventricular end-systolic volume (LVESV) >15% (odds ratio [OR] 1.50, 95% confidence interval [CI] 1.05–2.13, *P* = .025) and absolute reduction in LVESV (standardized mean difference [SMD] -0.25, 95% CI -0.43 to -0.08, *P* = .005) with guidance. CMR (OR 55.3, 95% CI 4.7–656.9, *P* = .002), electrical (OR 17.0, 95% CI 2.9–100, *P* = .002), multimodality imaging (OR 4.47, 95% CI 1.36–14.7, *P* = .014), and hemodynamic guidance (OR 1.29–28.0, *P* = .02) were significant in reducing LVESV >15%. Only STE demonstrated a significant reduction in absolute LVESV (SMD -0.38, 95% CI -0.68 to -0.09, *P* = .011]. CMR had the highest probability of improving clinical response (OR 17.9, 95% CI 5.14–62.5, *P* < .001).

**Conclusion:**

Overall, guidance improves CRT outcomes. STE and multimodality imaging provided the most reliable evidence of efficacy. Wide CIs observed for results of CMR guidance suggest more powered studies are required before a clear ranking is possible.

**Key Findings**

▪ Overall guidance and accurately placing the left ventricular lead in the optimal myocardial segment results in improved response to cardiac resynchronization therapy.
▪ Speckle-tracking echocardiography and multimodality imaging provided the most reliable evidence of efficacy in improving cardiac resynchronization therapy response. Cardiovascular magnetic resonance guidance was the most efficacious; however, wide confidence intervals and indirect evidence suggest approaching this with caution.
▪ Ranking superiority of guidance modalities remains difficult, and more appropriately powered studies are required.

## Introduction

Cardiac resynchronization therapy (CRT) is an effective treatment for patients with heart failure and electrical dyssynchrony characterized by left bundle branch block; however between 30% and 50% fail to derive benefit. CRT nonresponse is multifactorial; however, placement of the left ventricle (LV) pacing lead in the optimal position is considered integral to this.^1^ Although it is established that an apical position of the LV lead should be avoided, the best location across the LV axis is less certain.^2^ Evidence has demonstrated that a position away from scar^3^ at the point of latest mechanical activation (LMA) may significantly determine response.^4^ The targeting of the optimal LV lead position through a variety of guidance techniques has been investigated; however, with the increasing use of image-fusion technology^5^ and number of randomized trials being undertaken with more advanced imaging techniques,^6^ the need for an updated evaluation of the data is needed. A recently published meta-analysis by Hu and colleagues^7^ was limited to evaluating only image guidance and included both randomized and nonrandomized studies. This was limited by heterogenous study designs and excluded non-image-based guidance techniques.^7^ A network meta-analysis (NMA) was deemed appropriate to allow comparative assessment of different guidance modalities and potentially direct clinicians to which guidance modalities derived most benefit for patients.

## Methods

### Literature search

The systematic review and meta-analysis were conducted in accordance with the PRISMA guidelines (Supplemental Table S1).^8^ We systematically reviewed the relevant literature by searching EMBASE, CENTRAL, and MEDLINE databases from inception to June 2021 without language restriction. The Quality of Reporting of Meta-Analyses statement^9^ and the empiric study by McAuley and colleagues^10^ indicate the exclusion of unpublished studies produces a systematic positive bias, and therefore “gray literature” in the form of poster presentations, unpublished data from Cochrane Reviews or other meta-analyses, conference abstracts, and preprints were included. In addition, references of relevant literature were searched. The following keywords were used for search: (("guide") OR ("guided") OR ("guidance")) AND (("cardiac resynchronisation therapy") OR ("cardiac resynchronization therapy") OR ("LV lead") OR ("left ventricular lead")).

### Selection criteria

We included all eligible randomized studies that met the following inclusion criteria: (1) CRT-pacemaker/defibrillator (CRT-P/D), (2) heart failure with a QRSd >120 ms and LV ejection fraction <35%, (3) human studies only, and (4) minimum of 6 months mean follow-up. For each study, the following efficacy endpoints were evaluated: (1) echocardiographic volumetric response (change in left ventricular end-systolic volume [LVESV]) and (2) symptomatic response (as heterogenous reporting of this was expected, a change in Clinical Composite Score ≥1 or NYHA class ≥1 represented response). Change in LV ejection fraction was not included as an efficacy endpoint, as variable reporting of this outcome was anticipated.

### Data extraction

All data from included studies were independently extracted and assessed for further analysis by 2 reviewers (N.W. and V.S.M.). Any discrepancies were resolved through a third reviewer (M.K.E.). From each study, relevant information was extracted and tabulated. Study characteristics are reported. Modalities were split by whether only 1 imaging modality was used in the form of cardiac magnetic resonance (CMR), speckle-tracking echocardiography (STE), single-photon emission computed tomography (SPECT), cardiac computed tomography, or a combination of these, which was defined as “multimodality imaging.” Additional modalities evaluated included acute hemodynamic response (AHR) and electrophysiological guidance.

### Pairwise meta-analysis

A pairwise meta-analysis was performed in studies comparing an advanced guidance technique to fluoroscopic guidance (standard of care). Intention-to-treat data were used for evaluating endpoints from included studies whenever possible. The 95% confidence intervals (CIs), odds ratios (OR), and standardized mean difference (SMD) were computed for categorical and continuous variables, respectively. To estimate prespecified efficacy endpoints of continuous data, only those publications that contained both baseline and follow-up means ± standard deviations were used. A random-effects meta-analytical approach was applied to all analyses. Heterogeneity was considered low, moderate, and high for I^2^ values of <30%, 30%–60%, and >60%, respectively.^11^ Subgroup analyses were performed according to the type of guidance method. All analyses were performed using R version 1.3.1093 with the “metafor” package.^12^

### Network meta-analysis

The NMA was performed using the generic inverse variance method with the “netmeta” statistical package in R version 1.3.1093.^13^ Random-effects meta-analysis was reported, and inconsistency was evaluated with Cochran’s Q.^12^ Inconsistency between direct and indirect estimates was checked using “node splitting.”^14^ A significance level of .05 and CIs were used for testing, and all testing was 2-tailed. Rank scores with probability ranks of different treatment groups were calculated.^15^ These statistics are used to measure the extent of certainty that a treatment is better than another treatment (ranks closest to 1, being best), averaged over all competing treatments. Risk of bias for the individual studies was performed with the Cochrane Risk of Bias 2 tool.^16^ Publication bias was assessed by funnel plot and Egger’s test for each network analysis where ≥10 studies were included. The robustness of the inclusion of different patient subgroups and “grey literature” were tested by a sensitivity analysis.

## Results

A total of 1458 unique records were identified through the searches. After screening, 12 met inclusion criteria (Supplemental Figure S1). In total, 1977 patients were enrolled in the studies, with 1864 patients included in the final follow-up analyses (guidance arm, n = 1096; fluoroscopic arm, n = 768). In each guidance arm the following number of patients and studies were included: (1) AHR (n = 139, 1 study); (2) STE (n = 322, 3 studies); (3) electric (n = 273, 4 studies); (4) multimodality (n = 231, 4 studies); (5) CMR (n = 44, 1 study); (6) SPECT (n = 87, 1 study); (7) fluoroscopic (n = 768, 10 studies). Mean follow-up ranged from 6 to 24 months. Graphical risk-of-bias assessment is reported (Supplemental Figure S2). Relevant funnel plot assessing for publication bias in shown in Supplemental Figure S3.

### Study characteristics

Baseline demographics of included studies are summarized in Table 1. Three studies^17^^,^^18^ employed STE to identify the LMA and surrogates from circumferential strain imaging of viable myocardium in a mixed etiology group.^17^^–^^19^All multimodality imaging studies used STE to identify LMA alongside additional imaging modalities to identify scar, with 3 studies using computed tomography to identify coronary sinus (CS) anatomy.^20^, ^21^, ^22^ Stephansen and colleagues^22^ compared multimodality imaging to local LV electrical delay by measuring intrinsic left ventricular (LV) electric delay (QLV) in the basal, mid, and apical positions to identify the optimal LV lead position. Cannizzaro and colleagues^23^ used STE to identify LMA and scar transmurality derived from CMR imaging in an ischemic population. Kočková and colleagues^24^ used CMR to identify LMA using Tagging FLASH sequences^25^ outside of >50% scar and compared this to optimal site by QLV derivation. Zou and colleagues^26^ used SPECT to measure dyssynchrony using phase polar maps and myocardial perfusion uptake as a surrogate of viable myocardium. The optimal position was displayed using 3D LV surfaces alongside fluoroscopic images intraprocedurally. Singh and colleagues^27^ used QLV to guide LV lead placement in patients with right bundle branch block only. Sipal and colleagues^28^ aimed to implant the LV lead at the site with the narrowest biventricular-paced QRSd interprocedurally using surface electrocardiography. Finally, Sohal and colleagues^29^ identified the CS branch with the greatest AHR as derived by LV dP/dT_max_ as the optimal lead position. Baseline characteristics of study participants are reported in Table 2.

**Table 1:** Baseline demographics of included studies

Author, year	Study name	Guidance modality evaluated	Participants analyzed in each arm of study	Mean age ± SD	Ischemic etiology (%)	Male (%)	Mean LVEF ± SD or median (IQR) (%)	LVESV ± SD or median (IQR) (mL)	LBBB (%)	QRSd ± SD or median (IQR) (ms)	Follow-up (mo)
Sohal, 2021	RADI-CRT	*All participants*	278	70.8 ± 10.9	54%	74%	NR	NR	61%	NR	6
		AHR	139	71.1 ± 9.9	50%	70%	28 ± 5	129 ± 48	58%	161 ± 23	
		Anatomical	139	72.3 ± 10.5	57%	78%	29 ± 6	119 ± 44	64%	157 ± 23	
Saba, 2012	STARTER	*All participants*	187	NR	NR	NR	27 ± 6	140 ± 61	NR	NR	12
		STE	110	66 ± 11	58%	70%	26 ± 6	140 ± 59	NR	157 ± 27	
		Anatomical	77	67 ± 13	67%	78%	26 ± 7	144 ± 63	NR	162 ± 27	
Zou, 2019	GUIDE-CRT	*All participants*	177	NR	NR	NR	NR	NR	NR	NR	6
		SPECT	87	NR	NR	NR	NR	NR	NR	NR	
		Anatomical	90	NR	NR	NR	NR	NR	NR	NR	
Borgquist, 2020	CRT Clinic	*All participants*	102	68 ± 8	46%	73%	23 ± 11	167 (134–196)	74%	170 ± 19	47 ± 21
		Multimodality	53	67 ± 8	42%	74%	23 ± 10	172 (135–196)	74%	171 ± 16	
		Anatomical	49	70 ± 8	51%	73%	23 ± 12	163 (130–196)	74%	169 ± 22	
Stephansen, 2019	ElectroCRT	*All participants*	113	NR	NR	NR	NR	NR	NR	NR	6
		Multimodality	59	72 ± 8	53%	74%	29 ± 8	142 ± 56	100%	170 ± 17	
		Electrical	54	70 ± 10	47%	73%	31 ± 8	132 ± 54	100%	169 ± 23	
Sipal, 2018	NA	*All participants*	80	65.05 ± 9.05	40%	62.50%	NR	NR	100%	NR	6
		Electrical	40	64.45 ± 8.88	35%	60%	21.05 ± 4.83	132 (67–168)	100%	158.85 ± 13.93	
		Anatomical	40	65.65 ± 9.22	45%	65.00%	20.55 ± 5.02	191 (157–212)	100%	154.110 ± 13.50	
Kočková, 2018	CMR-CRT	*All participants*	95	NR	NR	NR	NR	NR	NR	NR	24
		CMR	44	64 ± 12	39%	70.00%	28 ± 7	133 ± 51	91%	165 ± 14	
		Electrical	51	64 ± 9	35%	67%	27 ± 7	155 ± 70	80%	165 ± 17	
Cannizzaro, 2015†	NA	*All participants*	90	64 ± 11	100%	68.80%	NR	NR	NR	NR	6
		Multimodality	30	NR	NR	NR	NR	NR	NR	NR	
		Anatomical	60	NR	NR	NR	NR	NR	NR	NR	
Khan, 2012	TARGET	*All participants*	207	NR	NR	NR	NR	NR	NR	NR	6
		STE	103	72 (65/76)	56%	77.00%	23 (19/28)	152 (118–183)	NR	157 (148–170)	
		Anatomical	104	72 (64/80)	56%	80%	24 (18/29)	149 (130–176)	NR	159 (146–170)	
Sommer, 2015	ImagingCRT	*All participants*	182	NR	NR	NR	NR	NR	NR	NR	6
		Multimodality	89	71 ± 9	52%	78%	25 ± 6	190 ± 70	100%	167 ± 22	
		Anatomical	93	71 ± 9	47%	80%	24 ± 6	198 ± 69	100%	165 ± 22	
Glickson, 2019†	Raise CRT	*All participants*	162	NR	100%	NR	NR	NR	NR	NR	12
		STE	109	69.7 ± 8.5	100%	96%	30.0 ± 8.5	28.5 ± 8	72%	155.2 ± 19.1	
		Anatomical	53	71.2 ± 8.4	100%	94%	NR	NR	77%	154.5 ± 17.4	
Singh, 2020	ENHANCE CRT	*All participants*	191	65.1 ±12.5	59.70%	80.20%	24.8 ± 7.1	NR	0%	NR	12
		Electrical	128	65.7 ± 12.1	62.10%	83.20%	24.8 ± 7.1	NR	0%	NR	
		Anatomical	63	64 ± 13.3	54.90%	74.40%	25.8 ± 7	NR	0%	NR	

AHR = acute hemodynamic response; CMR = cardiac magnetic resonance; CT = cardiac computed tomography; NA = not applicable; NR = not recorded; SPECT = single-photon emission computed tomography; STE = speckle-tracking echocardiography.

† Abstract only (not fully published results).

**Table 2:** Characteristics of included studies

Author, year, study name	Trial registration number	No. of participants enrolled (no. analyzed in final study)	Modalities compared	Follow-up time	Inclusion criteria	Study design	Single or multicenter	Primary endpoint	Secondary endpoints	Specified programming considerations	Lead type used (quadripolar/bipolar) & model type
Sohal, 2021, RADI-CRT	NCT 01464502	281 (278)	AHR (dP/dT) vs anatomical	6 months	EHRA criteria for CRT – (NYHA class II–IV drug-refractory HF, LV ejection fraction ≤ 35%, and prolonged QRS > 120 ms)	2-group parallel RCT	Multicenter	LVESV >15%	CCS (NYHA change >1, PGA, HF hospitalization, mortality); MLWHF; procedure duration; procedural complications; LVEF change; BNP; 6MWT	AV synchronous biventricular fashion. (DDDBiV), or biventricular fashion (VVIBiV) if in AF.	48% quadripolar in the guidance group vs 53% quadripolar in the guidance arm
Saba, 2013, STARTER	NCT 00156390	187 (187)	Echo (speckle) vs anatomical	1.8 years	EHRA criteria for CRT	Prospective double-blind RCT	Single	Time to first hospitalization or death	CCS (composite of death, heart transplantation, or LV assist device implantation); LVEF change; change in LVESV	NR	NR
Zou, 2019, GUIDE-CRT	NCT 03125720	194 (177)	SPECT vs anatomical	6 months	EHRA criteria for CRT	Prospective, multicenter, RCT	Multicenter	LVESV >15%	LVEDV change; LVEF change	NR	NR
Borgquist 2020, CRT Clinic	NCT 01426321	102 (102)	Mutimodality (echo - speckle + SPECT + CMR) vs Anatomical	2 years	EHRA criteria for CRT	Prospective blinded (single-vs-double) RCT	single	LVESV>15%	NYHA Class change>1; Death or HF hospitalization within 2 years	NR	49% quadripolar in the guidance group vs 40% in the anatomical group
Stephansen 2019, ElectroCRT	NCT 02346097	122 (113)	Electrical (QLV) vs multimodality (SPECT & echo - speckle)	6 months	EHRA criteria for CRT, including patients for upgrade (RV QRS >180 ms, and age >40)	Single center, patient and assessor blinded, RCT	Single	LVEF increase	CCS (absence of death; HF hospitalization; improved NYHA; increased 6MWT)	Optimization of VV delay	Overall 94% had quadripolar group
Sipal 2018, NA	NR	80 (80)	Electrical (surface ECG) vs conventional	6 months	EHRA criteria for CRT	Prospective double-blind RCT	Single	LVESV >15%	Change in QRSd, NYHA, proBNP, LVEF, LVEDV, LVESV, fluoroscopic time	AV and VV delays optimized echo. DDD(R) mode to achieve AV synchronous BiV pacing.	NR
Kočková 2018, CMR-CRT	NR	99 (95)	CMR vs electrical (QLV)	2 years	EHRA criteria for CRT	Prospective RCT	Single	Composite of CV death, HF hospitalization, at 2 years	NYHA class ≥1 (NYHA response), LVED diameter reduction by 10%; BNP reduction by ≥30% (BNP response)	Empiric AV delay of 120 ms and VV simultaneous programming	Both quadripolar and bipolar leads used (proportions not recorded)
Cannizzaro 2015,† NA	NR	90 (NR)	Multimodality (CMR + echo (speckle)) vs anatomical	6 months	EHRA criteria for CRT. Only ischemic etiology	Prospective, 1:2. Unclear if randomized	NR	LVESV >15%	NR	NR	NR
Khan 2012, TARGET	ISRCTN 19717943	220 (207)	Echo (speckle) vs anatomical	6 months	EHRA criteria for CRT	Prospective RCT. Assessor blinded	Multicenter	LVESV >15%	NYHA functional class >1; all-cause mortality; CCS (all-cause mortality and heart failure–related hospitalization)	DDD mode (lower rate limit, 40) to achieve atrial synchronous biventricular pacing	NR
Sommer 2015, ImagingCRT	NCT 01323686	182 (182)	Multimodality (cardiac CT + SPECT + echo - speckle) vs anatomical	1.8 ± 0.9 years	EHRA criteria for CRT, including patients for upgrade (RV QRS >180 ms, and age >40)	Prospective single-center RCT	Single	Clinical nonresponse: >1 of the following after 6 months: (1) death, (2) heart failure hospitalization, or (3) no improvement in NYHA class and <10% increase in 6-min walk distance	LV remodeling; all-cause mortality and hospitalization owing to heart failure	NR	NR
Glickson 2019,† Raise CRT	NCT 01603706	172 (162)	Echo (speckle) vs anatomical	1 year	EHRA criteria for CRT. Only ischemic etiology	Single-center randomized 2:1 (intervention vs control)	Single	Reduction in LVESV	Combined clinical event rate (death or HF hospitalization); LVESV >15%; >5% LVEF; MWLHF questionnaire; 6MWT	NR	NR
Singh 2020, ENHANCE CRT	NCT 01983293	248 (191)	Electrical (QLV) vs anatomical	1 year	Standard CRT inclusion; however, only non-LBBB with QRSd >120 ms	Multicenter randomized 2:1 (intervention vs control)	Multicenter	CCS (NYHA functional classification, a patient global assessment, heart failure events, and cardiovascular death)	NR	NR	All patients had quadripolar lead (St Jude Medical)

AHR = acute hemodynamic response; AV = atrioventricular; BiV = biventricular; BNP = brain natriuretic peptide; CMR = cardiac magnetic resonance; CCS = Clinical Composite Score; CRT = cardiac resynchronization therapy; CT = computed tomography; EHRA = European Heart Rhythm Association; HF = heart failure; IV = intravenous; LBBB = left bundle branch block; LV = left ventricular; LVED = left ventricular end diastolic; LVEDV = left ventricular end diastolic volume; LVEF = left ventricular ejection fraction; LVESV = left ventricular end-systolic volume; MLWHF = Minnesota Living with Heart Failure; NYHA = New York Heart Association; PGA = Patient Global Assessment; RCT = randomized controlled trial; RV = right ventricle; SPECT = single-photon emission computed tomography; STE = speckle-tracking echocardiography; 6MWT = 6-minute walk test.

† Abstract only (not fully published results).

### Pairwise meta-analysis

**Figure 1:**
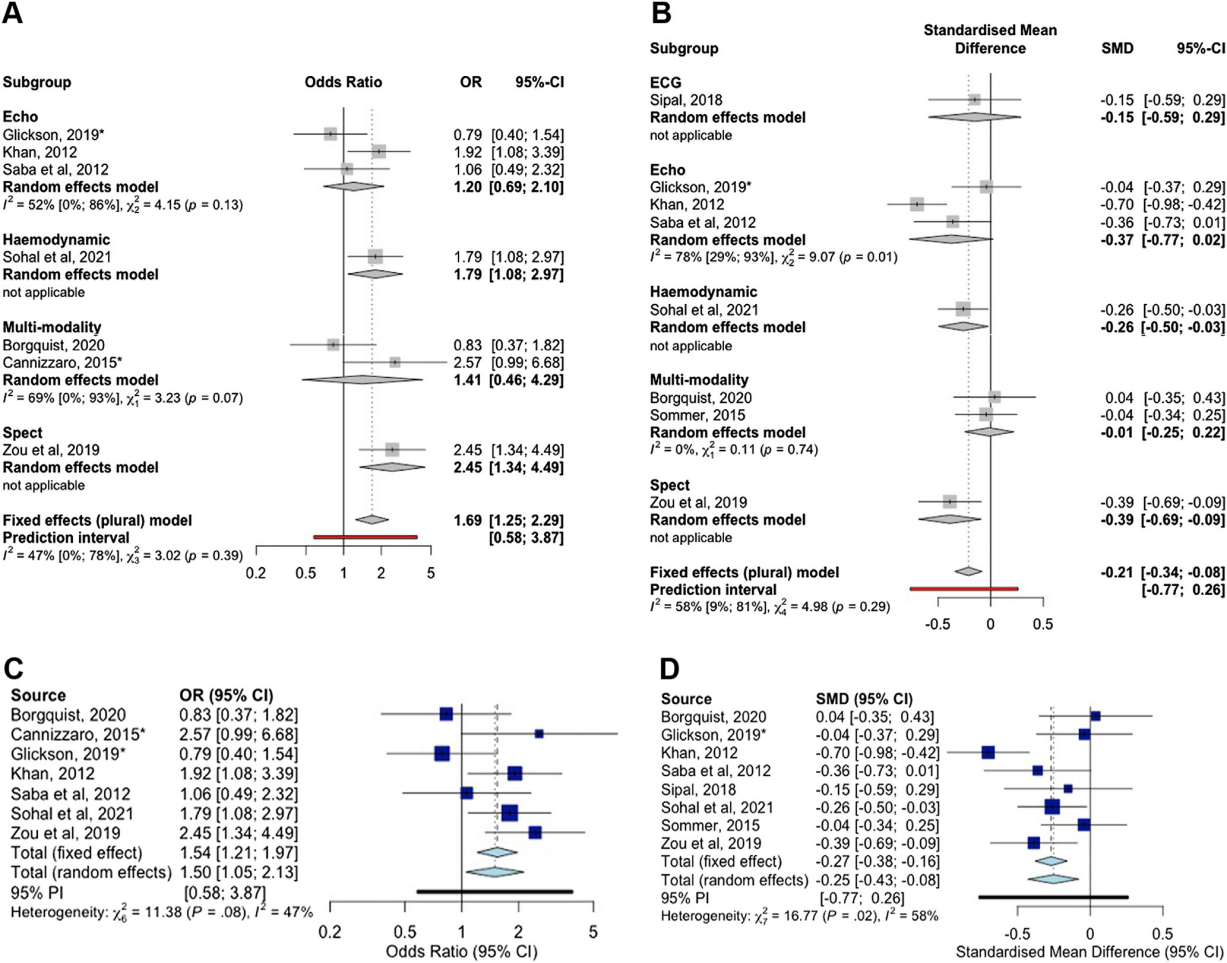
Forest plot displaying odds ratio (OR) of achieving marker of response dependent on guidance modality in comparison to anatomical guidance. **A:** OR of achieving reduction in left ventricular end-systolic volume (LVESV) >15%. **B:** Standardized mean difference (SMD) of absolute reduction in LVESV. **C:** Overall OR of achieving reduction in LVESV >15%. **D:** Overall standardized mean difference (SMD) of absolute reduction in LVESV.

Pairwise meta-analysis (Figure 1) evaluated studies comparing advanced guidance to fluoroscopic positioning. There was no significant improvement using STE with respect to LVESV >15% reduction (OR 1.20, 95% CI 0.69–2.10, *P* = .51, I^2^ = 52%), or absolute LVESV change (SMD -0.37, 95% CI -0.77 to 0.02, *P* = .065, I^2^ = 78%). Multimodality imaging did not demonstrate a significant improvement in LVESV >15% reduction (OR 1.41, 95% CI 0.46–4.29, *P* = .54, I^2^ = 69%) or absolute LVESV change (SMD -0.01, 95% CI -0.25 to 0.22, *P* = .91, I^2^ = 0%). There was a significant improvement in echo response by SPECT (OR 2.45, 95% CI 1.34–4.49, *P* = .004, I^2^ = not applicable [NA]) and absolute LVESV change (SMD -0.39, 95% CI -0.69 to -0.09, *P* = .01, I^2^ = NA). Similar findings were observed with respect to AHR guidance in LVESV >15% reduction (OR 1.79, 95% CI 1.08–2.97, *P* = .023, I^2^ = NA). Surface electrocardiographic guidance did not significantly reduce absolute LVESV (SMD -0.15, 95% CI -0.59 to 0.29, *P* = .58, I^2^ = NA). Overall, LV lead guidance resulted in a significant probability of reduction in LVESV >15% (OR 1.50, 95% CI 1.05–2.13, *P* = .025, I^2^ = 47%), and an observed absolute reduction in LV volumes (SMD -0.25, 95% CI -0.43 to -0.08, *P* = .005, I^2^ = 58%), when compared to fluoroscopic guidance.

### Network meta-analysis

#### Change in LVESV >15%

**Figure 2:**
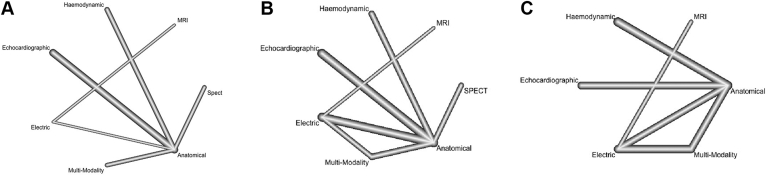
Network plots of eligible comparisons among the different guidance modalities. Lines represent direct comparisons, and the thickness of the lines indicates the number of studies comparing treatment pairs. Cardiac magnetic resonance imaging (MRI), electrical guidance (Electric), hemodynamic, single-photon emission computed tomography (Spect), and speckle-tracking echocardiography (Echocardiographic). **A:** Network plot of studies evaluating reduction in left ventricular end-systolic volume (LVESV) >15%. **B:** Network plot of studies evaluating absolute reduction in LVESV. **C:** Network plot of studies evaluating clinical response.

**Figure 3:**
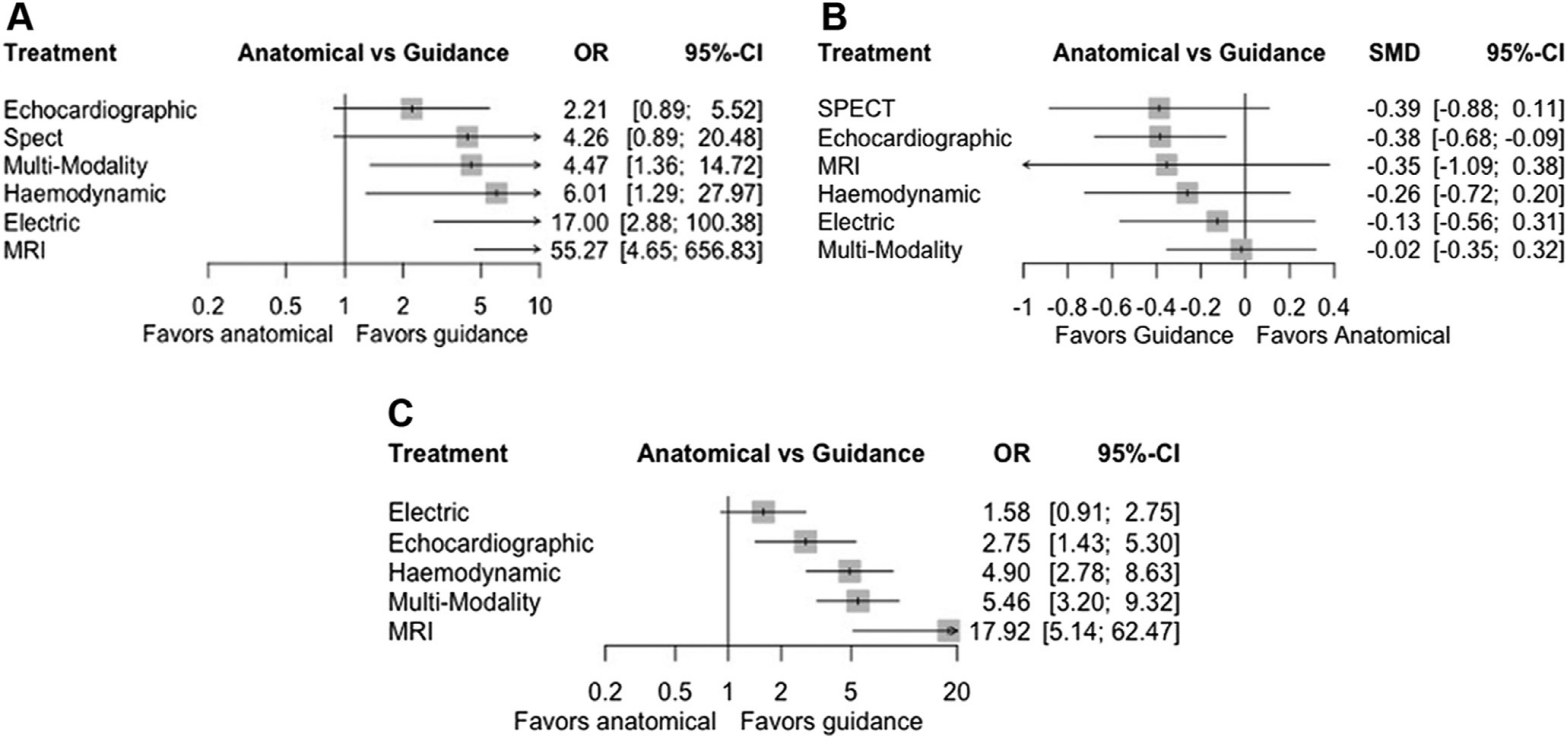
Network forest plots of different guidance modality comparisons for the following: **A:** Reduction in left ventricular end-systolic volume (LVESV) >15%; heterogeneity: I^2^ = 82%. **B:** Absolute reduction in LVESV; heterogeneity: I^2^ = 58%. **C:** Clinical response; heterogeneity: I^2^ = 69%. CI = confidence interval; MRI = cardiac magnetic resonance imaging; OR = odds ratio; SMD = standardized mean difference (SMD); Spect = single-photon emission computed tomography.

The evidence network is illustrated in Figure 2A and p-score ranking in Table 3. CMR (OR 55.3, 95% CI 4.7–656.9, *P* = .002), multimodality imaging (OR 4.47, 95% CI 1.36–14.7, *P* = .014), electrical (OR 17.0, 95% CI 2.9–100, *P* = .002), and hemodynamic (OR 6.01, 95% CI 1.29–28.0, *P* = .02) guidance demonstrated significant reduction in LVESV >15%, when compared to conventional fluoroscopic guidance. STE (OR 2.2, 95% CI 0.89–5.52, *P* = .089) and SPECT (OR 4.26, 95% CI 0.89–20.5, *P* = .07) were similarly on a direction favoring guidance (Figure 3A). Overall heterogeneity was high (I^2^ = 82%).

**Table 3:** Probability rank scores

LVESV >15%		Absolute LVESV reduction		Clinical response	
Modality	P-score	Modality	P-score	Modality	P-score
CMR	0.9589	STE	0.744	CMR	0.9868
Electric	0.7729	SPECT	0.7144	Multimodality	0.7163
dP/dT	0.5584	CMR	0.6581	dP/dT	0.6651
Multimodality	0.4769	dP/dT	0.5719	STE	0.4106
SPECT	0.4592	Electric	0.3917	Electric	0.2106
STE	0.2571	Multimodality	0.2335	Anatomical	0.0107
Anatomical	0.0166	Anatomical	0.1864	-	-

Rank scores with probability ranks of different guidance modalities cardiac magnetic resonance imaging (CMR), electrical guidance, hemodynamic (dP/dT), single-photon emission computed tomography (SPECT), and speckle-tracking echocardiography (STE). Ranks closest to 1 indicate the probability that the treatment group leads to greatest favorable outcome.

LVESV = left ventricular end-systolic volume.

#### Change in absolute LVESV

The evidence network is illustrated in Figure 2B and p-score ranking in Table 3. Compared to fluoroscopic guidance, only STE guidance demonstrated significance in reducing absolute LVESV volume (SMD -0.38, 95% CI -0.68 to 0.09, *P* = .011). No other guidance modalities were close to demonstrating significant reductions in LVESV volumes. Overall heterogeneity was moderate (I^2^ = 58%). A netsplit forest plot is shown in Supplemental Figure S4A.

#### Clinical response

The evidence network is illustrated in Figure 1C and p-score ranking in Table 3. Compared to fluoroscopic guidance, CMR (OR 17.9, 95% CI 5.14–62.5, *P* < .001), hemodynamic (OR 4.90, 95% CI 2.78–8.63, *P* < .001), multimodality imaging (OR 5.46, 95% CI 3.20–9.32, *P* < .001), and STE guidance (OR 2.75, 95% CI 1.43–5.30, *P* = .003] were significantly favorable with respect to clinical response. Only electrical guidance did not reach significance at improving clinical response (OR 1.51, 95% CI 0.91–2.75, *P* = .10). Overall heterogeneity was high (I^2^ = 69%). A netsplit forest plot is shown in Supplemental Figure S4B.

### Sensitivity analyses

Sensitivity analyses (Supplemental Figures S5 and S6) were performed excluding studies that compared specific and mixed etiology of heart failure patient populations; these were (1) non–left bundle branch block QRS morphology and (2) ischemic etiology only. Excluding the abstracts by Cannizzaro and colleagues and by Glikson and colleagues resulted in no differences in the odds ratios and significance of results with regard to the prespecified endpoints. As there was only 1 relatively small study evaluating CMR only,^24^ a sensitivity analysis was performed to test the stability of the results excluding this CMR-only study. The full results of the sensitivity analysis (Supplemental “sensitivity analysis”) support the inclusion of these specific groups in the meta-analysis.

### Other clinical outcomes

The Minnesota Living with Heart Failure questionnaire was assessed in 5 studies.^18^^,^^19^^,^^21^^,^^27^^,^^29^ Of these, only the ENHANCE CRT (guidance: 17.5 ± 26.4 vs conventional arm: 14.8 ± 20.8, *P* < .001)^27^ and TARGET study (guidance: 61 ± 76 vs conventional: 16 ± 19, *P* = .024)^18^ demonstrated a significant improvement with guidance. The 6-minute walk test was reported in 4 studies,^18^^,^^19^^,^^21^^,^^29^ with the RADI CRT (guidance: 68 ± 77 vs conventional: 43 ± 98, *P* = .02) and TARGET study (guidance: 61 ± 76 vs conventional: 38 ± 76, *P* = .011)^18^ demonstrating significant improvement. Of those reporting heart failure hospitalization and mortality, only 2 studies reported these separately rather than as part of a composite score.^17^^,^^27^ Saba and colleagues^17^ identified 30 deaths overall (guidance: 15 vs conventional: 15, *P* = .19), and 37 heart failure hospitalizations (16 vs 21, *P* = .049) and Singh and colleagues^27^ identified 39 subjects (26 in QLV arm and 13 in the conventional arm) who had heart failure events (8 cardiac deaths and 31 heart failure hospitalizations, *P* = .63).

## Discussion

Our main findings are as follows: (1) Overall guidance and accurately placing the LV lead in the optimal myocardial segment results in improved response to CRT. (2) STE and multimodality imaging provided the most reliable evidence of efficacy in improving CRT response. CMR guidance was the most efficacious; however, wide confidence intervals and indirect evidence suggest approaching this evidence with caution. (3) Ranking superiority of guidance modalities remains difficult and more appropriately powered studies are required.

### Type of guidance modality

One of the major determinants of CRT response is LV lead position with respect to myocardial scar distribution.^30^ This has the added advantage of potential reduced arrhythmogenicity.^31^ Another major determinant of response is placement of the LV lead at the latest electrically activated area—a surrogate of which is LMA.

The NMA identified that all guidance modalities were more efficacious than fluoroscopic guidance, with CMR guidance being most efficacious; however, the CMR evidence should be interpreted with caution, as it involved 1 study of 99 patients.^32^ AHR guidance also significantly improved response; however, this was from a single study,^29^ albeit with a higher number of patients (n = 281), and so can be interpreted with more certainty. Multimodality imaging significantly improved response and included 4 studies of 487 patients with narrower CIs, suggesting a greater certainty of the validity of these results.

Electrical guidance was not effective in the clinical response marker; however, this may be because most participants in these studies were from the ENHANCE-CRT study,^27^ which only included patients with right bundle branch block. STE was the only modality that was able to detect a significant absolute reduction in LVESV, which may be representative of the largest number of participants in the studies using this guidance modality (n = 322). In summary, the strongest evidence for guidance came from STE and multimodality imaging.

Overall, use of the NMA technique allowed to obtain mixed estimates. As consistency was found between direct and mixed estimates results, our guidance modalities’ effect size assessment is robust (Supplemental Figure S4).

### Ischemic vs nonischemic etiology

Cannizzaro and colleagues^23^ and Glikson and colleagues^19^ evaluated the role of guidance in an exclusively ischemic population, Cannizzaro finding a significant improvement in CRT response as defined by a reduction in LVESV >15% (73% vs 52%, *P* = .045), whereas Glikson identified no significant difference (48% vs 53%, *P* = NS). In comparison, the RADI-CRT study demonstrated greater reverse remodeling in an ischemic subgroup in the pressure-wire guided arm (69% vs 49%, *P* = .02), but not in the nonischemic subgroup (81% vs 71%, *P* = .19). The TARGET study did not demonstrate significant reverse remodeling on multivariable analysis in an ischemic population (OR 1.54, 95% CI 0.69–3.43, *P* = .293); however, absence of scar at the LV lead pacing site did increase likelihood of response (OR 3.06, 95% CI 1.01–9.26, *P* = .048).^18^ This suggests an ischemic population may have the most benefit from avoiding scar and therefore from guidance use. The sensitivity analysis whereby the ischemic-only studies were excluded (Supplemental Figure S6B) suggests that ischemic patients presented a disproportionately greater challenge to improved outcomes.

### Accuracy of final LV lead location

Borgquist and colleagues^20^ evaluated outcomes based on whether the final LV lead was concordant to the optimal cardiac segment or in the adjacent segment and compared this to those with an LV lead in a distant segment. It was found that death or heart failure hospitalization was more likely in the distant LV lead group (*P* = .008).^20^ Saba and colleagues^17^ observed that pacing at optimal LV sites conferred significantly better CRT-D therapy–free survival rate compared to pacing remotely to the optimal site (HR = 0.51, 95% CI 0.28–0.90, *P* = .018). Khan and colleagues^18^ demonstrated that a concordant position was increased likelihood of echo reverse remodeling (OR 4.43, 95% CI 2.09–9.40, *P* = .009). Sommer and colleagues^21^ observed that lead placement in the priority CS branch resulted in lower rate of clinical nonresponse to CRT (25% vs 57%, *P* < .001). Zou and colleagues^26^ also demonstrated that concordance achieved significantly greater LVESV reduction (44.8 ± 54.2 mL vs 19.4 ± 74.3 mL, *P* = .024), and response rate (57.1% vs 35.0%; *P* = .025).

### Clinical and future perspective

These clinical trials have demonstrated that integrating guidance into CRT implantation is feasible; however, there continues to be reluctance to integrate guidance routuinely.^1^ Potential reasons include the additional cost, investigations, expertise, and equipment required. A proportion of patients may have poor image quality, or may not have the required coronary venous anatomy to reach the desired segment.^33^, ^34^, ^35^ This NMA has demonstrated that integrating STE is effective in detecting a significant absolute reduction in LVESV. As echocardiography is part of the minimum dataset required pre-CRT,^33^ this could be fundamental to image guidance without burdensome additional equipment.

It must be noted that final LV lead position identification was based on fluoroscopy images in the studies, which is known to be poorly reproducible.^36^ More advanced fusion image–based guidance systems are currently used in an investigational setting and have the potential to identify the target LV segment more precisely in real time during implantation.^5^ Such increased precision may derive further benefit from guidance. Large, ongoing randomized trials will provide greater insights into this technology’s effectiveness.^37^

### Limitations

The current NMA has important limitations. There were multiple different measures of CRT outcome and follow-up duration was not consistent across studies; therefore, the most common outcome markers were evaluated. Patients and outcome assessors were not uniformly masked to whether they were in the intervention group, which may introduce treatment and observer bias. Not all studies were 1:1 randomized. In some of the network arms there was 1 study with smaller numbers of patients included, notably evaluating CMR only. This accounts for the wide confidence intervals observed and suggests further clinical trials will increase confidence of their efficacy. Specific patient populations were only recruited in some of the studies, which may reduce the reproducibility of these results in a general dyssynchronous heart failure population. Two studies were not fully published results; however, the inclusion of “gray literature” was to avoid selection and publication bias. Sensitivity analyses were performed to mitigate this risk, and these results demonstrate the robustness of the inclusion of these studies. Only 4 studies specified what proportion of quadripolar or bipolar lead was implanted; 3 used both, with no significant differences between the guidance and fluoroscopic group.^20^^,^^22^^,^^29^ In addition, only 5 studies identified type of programming specified postimplant and whether the device was optimized. The lack of consistency in programming and lead technology may affect the interpretation of these results. Finally, the data used were derived data published by the study authors, and therefore patient-level data were not used for the meta-analysis.

## Conclusion

This comprehensive analysis suggests that overall, guiding the LV led to the optimal myocardial segment results in better CRT response. Further evidence in the form of large, randomized studies will allow a more nuanced evaluation of which modality is best placed to guide optimal LV lead delivery, particularly in advanced imaging modalities such as CMR. Easily accessible, reproducible, and interpretable techniques are essential for widespread integration of guidance into routine clinical care.

## Supplementary data

Supplementary_Table

Clean_Supplemental Results

Clean_Supplementary_figures_revised
