# Supplementary material for: The role of guidance in delivering cardiac resynchronization therapy: A systematic review and network meta-analysis
Source: Heart Rhythm O2. 2022 Jul 20;3(5):482–92. doi: 10.1016/j.hroo.2022.07.005 (PMC9626880; doi:10.1016/j.hroo.2022.07.005)
Supplement: Clean_Supplemental Results [file mmc2.docx]

Sensitivity Analysis

Excluding the studies comparing only non-LBBB to a mixed QRS morphology population resulted in the exclusion of one study ^1^. The resultant evidence network is illustrated in supplementary figure S5A. Compared to fluoroscopic guidance, all guidance modalities, including electrical guidance, have shown significantly improved clinical response (supplementary figure 6A). This suggests that electrical guidance may be more beneficial in a LBBB population, and that the inclusion of this study did not affect the robustness of the overall NMA results.

Excluding the studies comparing only ischaemic populations to a mixed aetiology population resulted in the exclusion of two studies ^2,3^. The resultant evidence networks are illustrated in supplementary figure S5B. Compared to fluoroscopic guidance all guidance modalities except STE significantly improved clinical response; and all modalities except multi-modality imaging reduced LVESV>15% as assessed by odds ratios. CMR, electrical and multi-modality guidance did not result in a significant reduction in absolute LVESV. Across all three endpoints, the exclusion of the ischaemic only studies, guidance resulted in better outcomes of patients. By removing the studies by Glikson and Cannizaro, we have also excluded the abstracts. The removal of this grey literature and the results of the sensitivity analysis support the inclusion of grey literature in the initial analysis (supplementary figure S6B).

Removal of studies that utilised only CMR guidance, resulted in the exclusion of one study^4^. The resultant evidence networks are illustrated in supplementary figure S5C. By removing this study, all guidance modalities except for echocardiographic and SPECT guidance resulted in a significantly improved odds ratio of reducing LVESV>15%; only echocardiographic was able to detect a significant reduction in absolute LVESV; and all guidance modalities except for STE guidance resulted in an improvement in clinical response (supplementary figure S6C). This suggests the inclusion of this study did not affect the robustness of the NMA results.

References

1. Singh JP, Berger RD, Doshi RN, et al. Targeted Left Ventricular Lead Implantation Strategy for Non-Left Bundle Branch Block Patients: The ENHANCE CRT Study. *JACC: Clinical Electrophysiology*. 2020;6(9):1171-1181. doi:10.1016/j.jacep.2020.04.034

2. Glikson M, Golovchiner G, Swissa M, et al. Speckle tracking radial strain imaging-guided lead placement for improving response to crt in patients with ischemic cardiomyopathy. *Europace*. 2019;21(1):ii836‐. https://www.cochranelibrary.com/central/doi/10.1002/central/CN-01959585/full

3. Chiodi E, Ferrante Z, Giganti M. Cardiac resynchronization in ischemic heart failure patients : a comparison between therapy guided by cardiac magnetic resonance imaging and 2D-speckle tracking. 2015;(March). doi:10.1594/ecr2015/C-0190

4. Kočková R, Sedláček K, Wichterle D, et al. Cardiac resynchronization therapy guided by cardiac magnetic resonance imaging: A prospective, single-centre randomized study (CMR-CRT). *International Journal of Cardiology*. 2018;270:325-330. doi:10.1016/j.ijcard.2018.06.009
