# Supplementary material for: The role of guidance in delivering cardiac resynchronization therapy: A systematic review and network meta-analysis
Source: Heart Rhythm O2. 2022 Jul 20;3(5):482–92. doi: 10.1016/j.hroo.2022.07.005 (PMC9626880; doi:10.1016/j.hroo.2022.07.005)

**Supplement**

﻿**Figure S1:** PRISMA (Preferred Reporting Items for Systematic Reviews and Meta-Analyses) flow chart for literature search and study selection. Twelve studies were included in the final analysis.


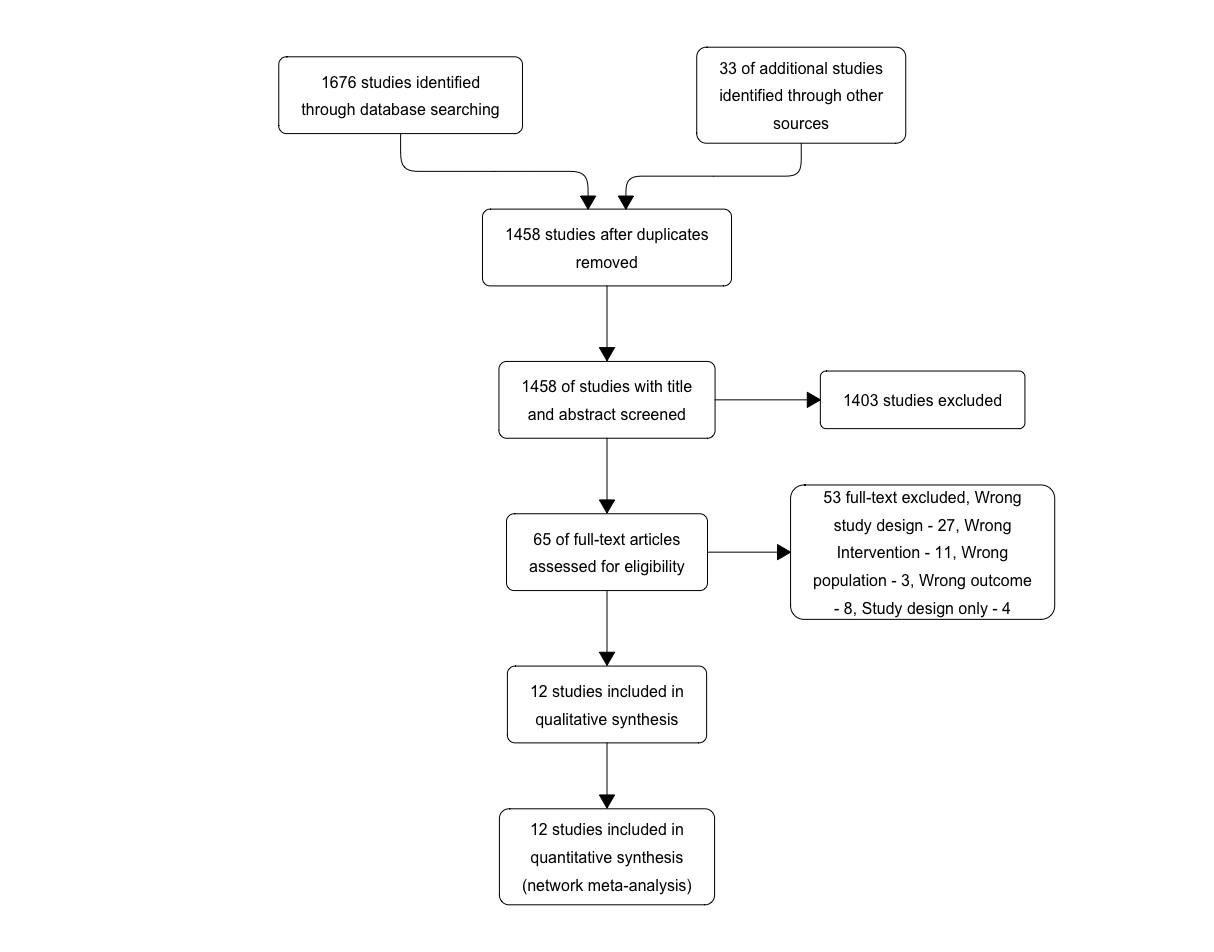


**Figure S2a:** Graphical risk of bias (RoB) by individual study

**Supplement Figure S2b:** Graphic RoB across studies

**Figure S3:** Funnel plot assessing for publication bias, based on 10 studies used in the network evaluating reduction in LVESV>15%.


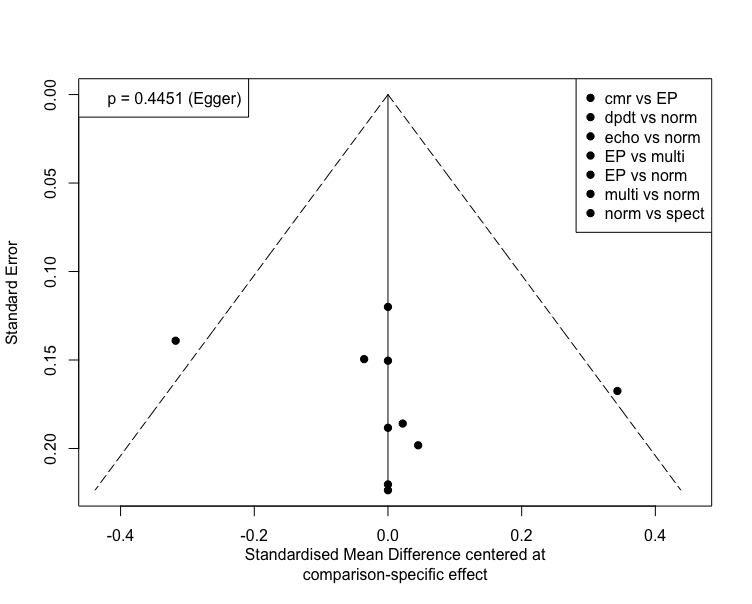


**Figure S4:** Net split forest plot.This splits our network estimates into the contribution of direct and indirect evidence, which allows to control for inconsistency in the estimates of individual comparisons..(A) Absolute reduction in LVESV (p=0.159); (B) Clinical Response (p=0.171).


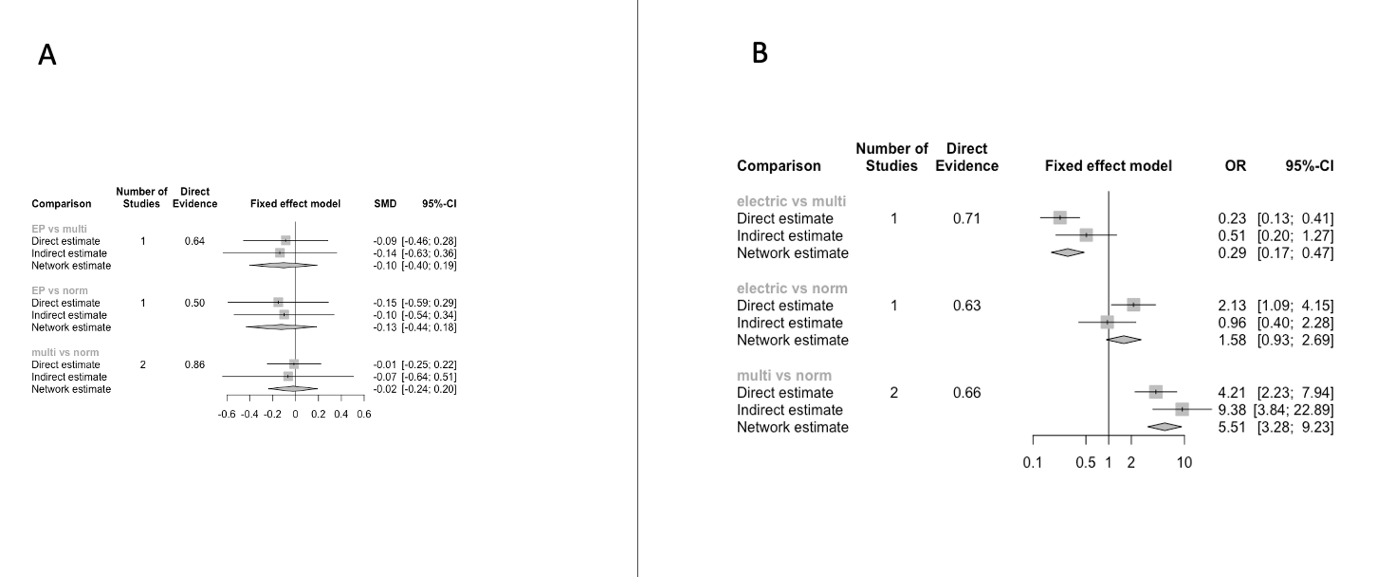


Figure S5: Network plots of eligible comparisons among the different guidance modalities of each sensitivity analysis. Lines represent direct comparisons, and the thickness of the lines indicates the number of studies comparing treatment pairs. cardiac magnetic resonance imaging (MRI), electrical guidance (electric), haemodynamic, single-photon emission computed tomography (SPECT), and speckle tracking echocardiography (echocardiographic).

Figure S5A: Sensitivity analysis excluding non-LBBB only studies. (I) Network plot of studies evaluating clinical response.


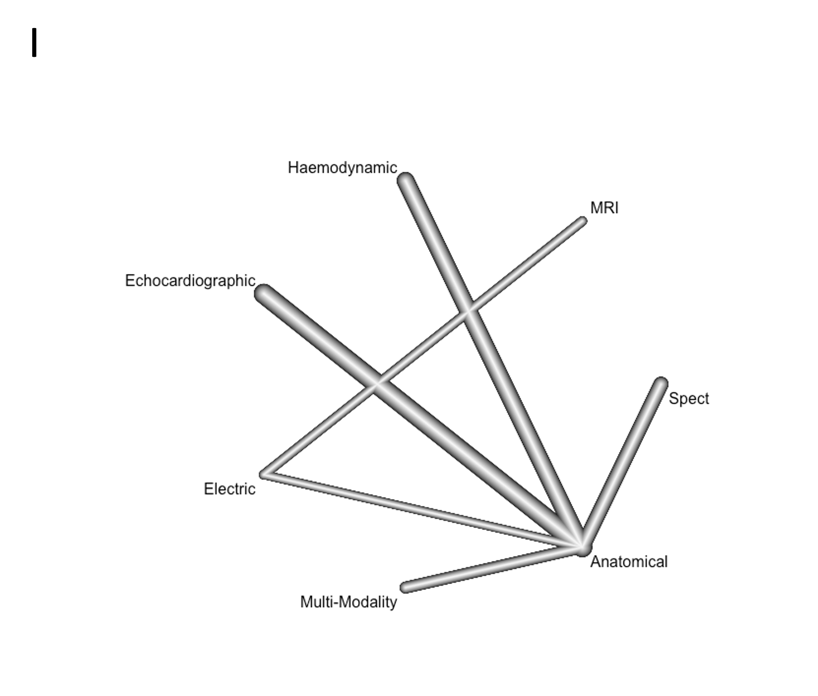


Figure S5B: Sensitivity analysis excluding ischemic only studies. (I) Network plot of studies evaluating reduction in left ventricular end systolic volume (LVESV) >15%.(II) Network plot of studies evaluating absolute reduction in LVESV.(III) Network plot of studies evaluating clinical response.


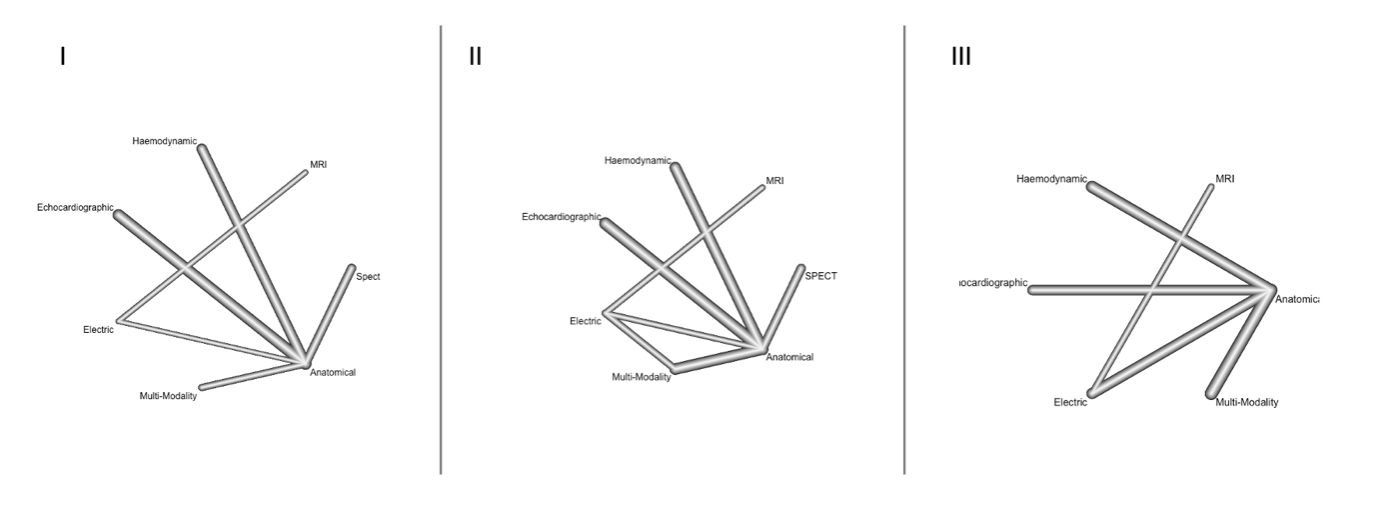


Figure S5C: Sensitivity analysis excluding MRI only studies. (I) Network plot of studies evaluating reduction in left ventricular end systolic volume (LVESV) >15%.(II) Network plot of studies evaluating absolute reduction in LVESV.(III) Network plot of studies evaluating clinical response.


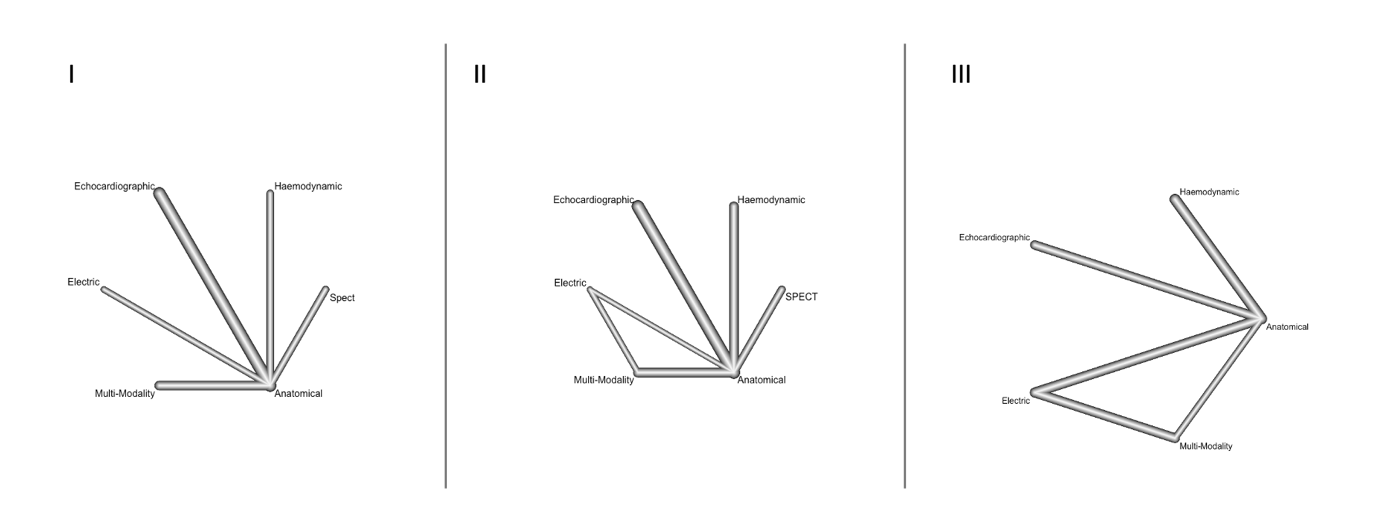


Figure S6: ﻿Network forest plots of different guidance modality comparisons. Cardiac magnetic resonance imaging (MRI), single-photon emission computed tomography (SPECT), odds ratio (OR), standardized mean difference (SMD), confidence interval (CI).

Figure S6A: Sensitivity analysis excluding non-LBBB studies only.(I) Reduction in LVESV >15%


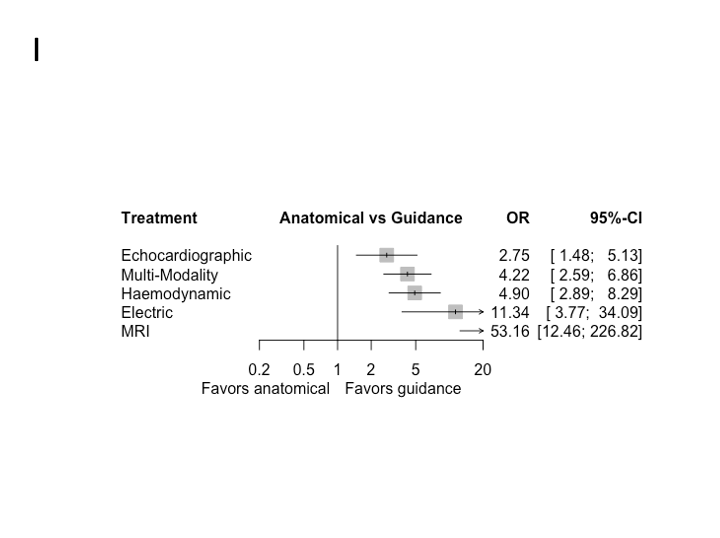


Figure S6B: Sensitivity analysis excluding ischemic only studies (as the ischemic only studies were also the only abstracts included in the sensitivity analysis, this figure also represents the results of a sensitivity analysis excluding “grey literature”).(I) Reduction in LVESV >15%; (II) Absolute reduction in LVESV; (III) Clinical Response.


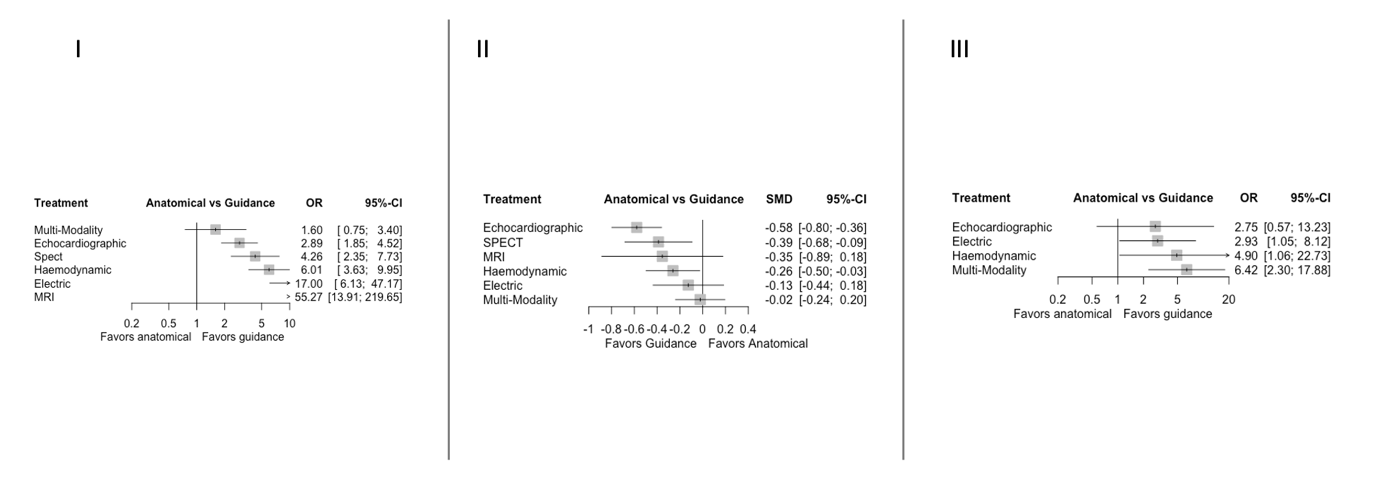


Figure S6C: Sensitivity analysis excluding MRI only studies.(I) Reduction in LVESV >15%; (II) Absolute reduction in LVESV; (III) Clinical Response.


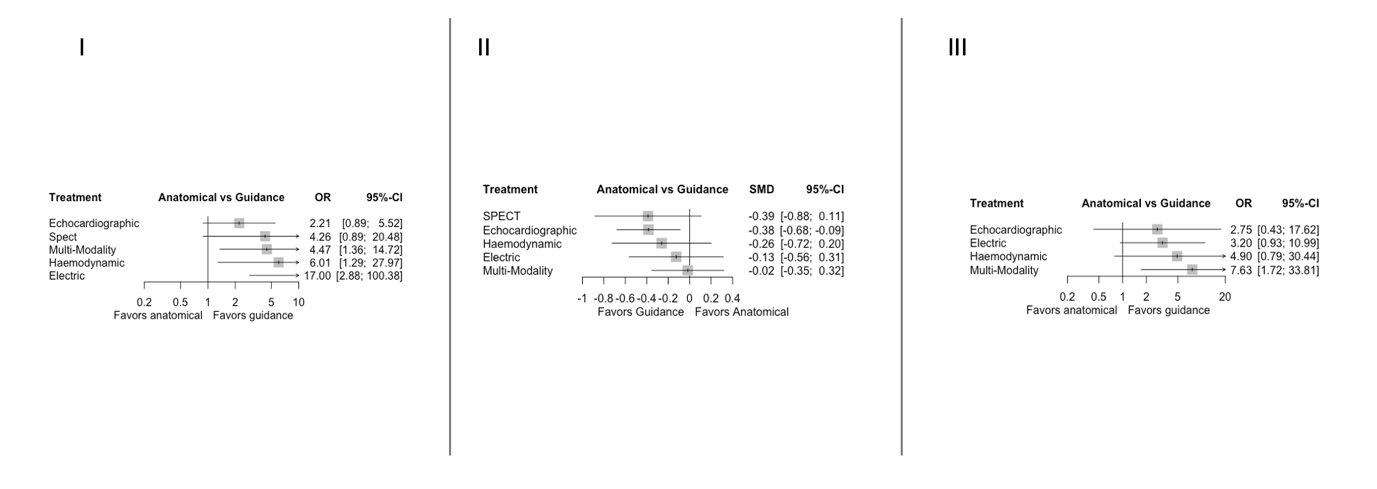

Supplement: Clean_Supplementary_figures_revised [file mmc3.docx]
